# 3-(2-Bromo­acet­yl)-6-fluoro-2*H*-chromen-2-one

**DOI:** 10.1107/S1600536811030960

**Published:** 2011-08-06

**Authors:** H. N. HarishKumar, Sudarshan Mahapatra, K. N. Venugopala, Kittappa Mariswamy Mahadevan

**Affiliations:** a Department of Post Graduate Studies and Research in Chemistry, School of Chemical Sciences Kuvempu University, Shankaraghatta, Karnataka 577 451, India; b Solid State and Structural Chemistry Unit, Indian Institute of Science, Bangalore 560 012, India

## Abstract

The non-H atoms of the title compound, C_11_H_6_BrFO_3_, are essentially coplanar (r.m.s. deviation for all non-H atoms = 0.074 Å). In the crystal, the molecules are linked by C—H⋯O and C—H⋯Br inter­actions.

## Related literature

For background to coumarins, see: Hooper *et al.*,(1982 ▶); Morris *et al.* (1971 ▶); Khalfan *et al.* (1987 ▶); Domagala *et al.* (1996 ▶); Eid *et al.* (1994 ▶).
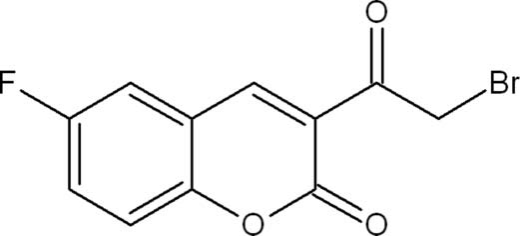


## Experimental

### 

#### Crystal data

C_11_H_6_BrFO_3_
*M* *_r_* = 285.06
Monoclinic, 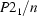

*a* = 4.0590 (5) Å
*b* = 11.7719 (13) Å
*c* = 21.608 (2) Å
β = 94.318 (10)°
*V* = 1029.6 (2) Å^3^
*Z* = 4
Mo *K*α radiation
μ = 3.99 mm^−1^
*T* = 293 K
0.30 × 0.20 × 0.10 mm

#### Data collection

Bruker SMART CCD area-detector
Absorption correction: multi-scan (*SADABS*; Sheldrick, 1996 ▶) *T*_min_ = 0.201, *T*_max_ = 0.506
10491 measured reflections
2007 independent reflections
1438 reflections with *I* > 2σ(*I*)
*R*_int_ = 0.036

#### Refinement

*R*[*F* ^2^ > 2σ(*F* ^2^)] = 0.029
*wR*(*F* ^2^) = 0.065
*S* = 0.95
2007 reflections
145 parameters
H-atom parameters constrained
Δρ_max_ = 0.21 e Å^−3^
Δρ_min_ = −0.37 e Å^−3^

### 

Data collection: *SMART* (Bruker, 1998 ▶); cell refinement: *SAINT* (Bruker, 1998 ▶); data reduction: *SAINT*; program(s) used to solve structure: *SHELXTL* (Sheldrick, 2008 ▶); program(s) used to refine structure: *SHELXL97* (Sheldrick, 2008 ▶); molecular graphics: *ORTEP-3* for Window (Farrugia, 1997 ▶); software used to prepare material for publication: *PLATON* (Spek, 2009 ▶).

## Supplementary Material

Crystal structure: contains datablock(s) global, I. DOI: 10.1107/S1600536811030960/bt5592sup1.cif

Structure factors: contains datablock(s) I. DOI: 10.1107/S1600536811030960/bt5592Isup2.hkl

Supplementary material file. DOI: 10.1107/S1600536811030960/bt5592Isup3.cml

Additional supplementary materials:  crystallographic information; 3D view; checkCIF report

## Figures and Tables

**Table 1:** Hydrogen-bond geometry (Å, °)

*D*—H⋯*A*	*D*—H	H⋯*A*	*D*⋯*A*	*D*—H⋯*A*
C3—H3⋯O3^i^	0.93	2.45	3.296 (3)	152
C7—H7⋯O2^ii^	0.93	2.52	3.425 (3)	164
C11—H11*A*⋯Br1^iii^	0.97	2.89	3.747 (3)	148

Symmetry codes: (i) 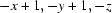; (ii) 
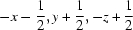
; (iii) 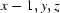.

**Fig. 1.**
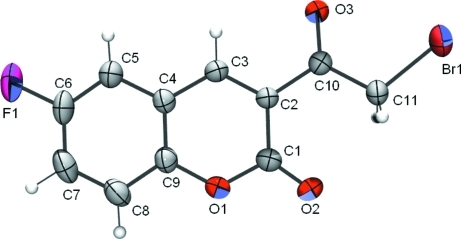
View of the title compound with the atom numbering scheme. Displacement ellipsoids for non-H atoms are drawn at the 50% probability level.

## supplementary crystallographic information

### Comment

Coumarine derivatives have potential application in the dye industry (Hooper *et al.*,1982 & Morris *et al.*, 1971), developing LASER dyes(Khalfan *et al.*,1987) and pharmaceutical industry for their antiviral activity (Domagala *et al.*,1996) and antimicrobial activity (Eid *et al.*, 1994). 3-Acetyl coumarins is found to be a major compound in the coumarine series and the title compound is a member of this family. The molecule forms well defined dimer *via* C—H···O intermolecular interaction through the center of inversion. The three dimensional packing motif in the title compound is built up of C—H···O and C—H···Br intermolecular interaction.

### Experimental

Synthesis of 3-Bromoacetyl-6-fluoro-2*H*-1-benzopyran-2-one: To a solution of compound 3-acetyl-6-fluoro-2*H*-1-benzopyran-2-one (206 mg, 1 mmol) in alcohol free chloroform (5 ml), bromine (173.8 mg, 1.1 mmol) in chloroform (2 ml) was added with intermittent shaking and warming. The mixture was heated for fifteen minutes on a water bath, cooled and filtered. The solid was washed with ether and crystallized from glacial acetic acid to yield 3-bromoacetyl-6-fluoro-2*H*-1-benzopyran-2-one.

Crystallization: Needle shape crsytals of 3-acetyl-6-fluoro-2*H*-1-benzopyran-2-one was obtained by dissolving in glacial aceic acid and warmed for few minutes in a 5 ml beaker. Then the total content was covered by paraffin film with few punches and kept for crystallization at room temperature.

### Refinement

All H atoms were positioned geometrically and refined using a riding model.

### Crystal data

**Table:**

C_11_H_6_BrFO_3_	*F*(000) = 560
*M**_r_* = 285.06	*D*_x_ = 1.839 Mg m^−^^3^
Monoclinic, *P*2_1_/*n*	Mo *K*α radiation, λ = 0.71073 Å
Hall symbol: -P 2yn	Cell parameters from 2007 reflections
*a* = 4.0590 (5) Å	θ = 3.3–26.0°
*b* = 11.7719 (13) Å	µ = 3.99 mm^−^^1^
*c* = 21.608 (2) Å	*T* = 293 K
β = 94.318 (10)°	Needle, yellow
*V* = 1029.6 (2) Å^3^	0.30 × 0.20 × 0.10 mm
*Z* = 4	

### Data collection

**Table:**

Bruker SMART CCD area-detector diffractometer	2007 independent reflections
Radiation source: Enhance (Mo) X-ray Source	1438 reflections with *I* > 2σ(*I*)
graphite	*R*_int_ = 0.036
Detector resolution: 16.0839 pixels mm^-1^	θ_max_ = 26.0°, θ_min_ = 3.3°
ω scans	*h* = −5→4
Absorption correction: multi-scan (*SADABS*; Sheldrick, 1996)	*k* = −14→14
*T*_min_ = 0.201, *T*_max_ = 0.506	*l* = −26→26
10491 measured reflections	

### Refinement

**Table:**

Refinement on *F*^2^	Primary atom site location: structure-invariant direct methods
Least-squares matrix: full	Secondary atom site location: difference Fourier map
*R*[*F*^2^ > 2σ(*F*^2^)] = 0.029	Hydrogen site location: inferred from neighbouring sites
*wR*(*F*^2^) = 0.065	H-atom parameters constrained
*S* = 0.95	*w* = 1/[σ^2^(*F*_o_^2^) + (0.0335*P*)^2^] where *P* = (*F*_o_^2^ + 2*F*_c_^2^)/3
2007 reflections	(Δ/σ)_max_ = 0.001
145 parameters	Δρ_max_ = 0.21 e Å^−^^3^
0 restraints	Δρ_min_ = −0.37 e Å^−^^3^

### Special details

**Table:**

Geometry. Bond distances, angles *etc*. have been calculated using the rounded fractional coordinates. All su's are estimated from the variances of the (full) variance-covariance matrix. The cell e.s.d.'s are taken into account in the estimation of distances, angles and torsion angles
Refinement. Refinement of F^2^ against ALL reflections. The weighted R-factor wR and goodness of fit S are based on F^2^, conventional R-factors R are based on F, with F set to zero for negative F^2^. The threshold expression of F^2^ > 2sigma(F^2^) is used only for calculating R-factors(gt) etc. and is not relevant to the choice of reflections for refinement. R-factors based on F^2^ are statistically about twice as large as those based on F, and R- factors based on ALL data will be even larger.

### Fractional atomic coordinates and isotropic or equivalent isotropic displacement parameters (Å^2^)

**Table:**

	*x*	*y*	*z*	*U*_iso_*/*U*_eq_	
Br1	0.29450 (7)	0.10699 (2)	−0.07092 (1)	0.0553 (1)	
F1	0.1896 (5)	0.78595 (14)	0.21546 (8)	0.0728 (7)	
O1	−0.1428 (4)	0.34429 (15)	0.16976 (8)	0.0450 (6)	
O2	−0.1904 (5)	0.19171 (16)	0.11181 (9)	0.0630 (8)	
O3	0.3915 (5)	0.34027 (16)	−0.01840 (9)	0.0606 (8)	
C1	−0.0789 (7)	0.2860 (2)	0.11695 (12)	0.0405 (9)	
C2	0.1133 (6)	0.3458 (2)	0.07253 (11)	0.0320 (8)	
C3	0.1926 (6)	0.4562 (2)	0.08252 (11)	0.0351 (9)	
C4	0.1121 (6)	0.5154 (2)	0.13648 (11)	0.0338 (8)	
C5	0.1933 (7)	0.6296 (2)	0.14854 (13)	0.0428 (10)	
C6	0.1139 (7)	0.6754 (2)	0.20335 (14)	0.0478 (10)	
C7	−0.0399 (7)	0.6145 (3)	0.24778 (13)	0.0531 (11)	
C8	−0.1208 (7)	0.5031 (3)	0.23639 (12)	0.0487 (10)	
C9	−0.0485 (6)	0.4551 (2)	0.18082 (11)	0.0372 (9)	
C10	0.2260 (6)	0.2882 (2)	0.01622 (12)	0.0362 (9)	
C11	0.1383 (7)	0.1654 (2)	0.00482 (12)	0.0417 (9)	
H3	0.30428	0.49474	0.05289	0.0421*	
H5	0.29859	0.67285	0.11987	0.0514*	
H7	−0.08758	0.64873	0.28485	0.0637*	
H8	−0.22291	0.46050	0.26572	0.0585*	
H11A	−0.10000	0.15723	0.00318	0.0500*	
H11B	0.23180	0.12039	0.03940	0.0500*	

### Atomic displacement parameters (Å^2^)

**Table:**

	*U*^11^	*U*^22^	*U*^33^	*U*^12^	*U*^13^	*U*^23^
Br1	0.0651 (2)	0.0464 (2)	0.0556 (2)	−0.0025 (2)	0.0125 (2)	−0.0186 (2)
F1	0.1021 (14)	0.0435 (11)	0.0734 (12)	−0.0040 (10)	0.0097 (10)	−0.0273 (9)
O1	0.0634 (12)	0.0387 (11)	0.0351 (10)	−0.0073 (10)	0.0176 (9)	−0.0014 (9)
O2	0.0971 (16)	0.0386 (13)	0.0576 (13)	−0.0262 (12)	0.0342 (12)	−0.0060 (10)
O3	0.0977 (16)	0.0382 (11)	0.0506 (12)	−0.0227 (12)	0.0377 (12)	−0.0104 (10)
C1	0.0487 (17)	0.0389 (17)	0.0346 (15)	−0.0033 (14)	0.0086 (13)	0.0010 (13)
C2	0.0372 (15)	0.0297 (14)	0.0297 (14)	−0.0041 (12)	0.0070 (12)	0.0004 (11)
C3	0.0406 (15)	0.0320 (16)	0.0331 (15)	−0.0054 (12)	0.0065 (12)	0.0017 (11)
C4	0.0361 (14)	0.0329 (15)	0.0324 (14)	0.0025 (12)	0.0027 (12)	−0.0025 (12)
C5	0.0499 (17)	0.0359 (17)	0.0433 (16)	−0.0014 (13)	0.0075 (13)	−0.0040 (12)
C6	0.0550 (18)	0.0336 (17)	0.0540 (19)	0.0068 (15)	−0.0013 (16)	−0.0144 (14)
C7	0.063 (2)	0.055 (2)	0.0413 (17)	0.0138 (17)	0.0044 (15)	−0.0154 (15)
C8	0.0570 (19)	0.0529 (19)	0.0376 (16)	0.0047 (16)	0.0123 (14)	−0.0051 (14)
C9	0.0416 (16)	0.0338 (16)	0.0363 (15)	0.0039 (13)	0.0037 (13)	−0.0037 (12)
C10	0.0407 (15)	0.0321 (16)	0.0361 (15)	−0.0031 (12)	0.0053 (12)	−0.0013 (12)
C11	0.0477 (16)	0.0343 (16)	0.0446 (16)	−0.0053 (14)	0.0142 (13)	−0.0095 (12)

### Geometric parameters (Å, °)

**Table:**

Br1—C11	1.926 (3)	C5—C6	1.362 (4)
F1—C6	1.358 (3)	C6—C7	1.384 (4)
O1—C1	1.373 (3)	C7—C8	1.370 (5)
O1—C9	1.375 (3)	C8—C9	1.379 (4)
O2—C1	1.201 (3)	C10—C11	1.505 (3)
O3—C10	1.209 (3)	C3—H3	0.9300
C1—C2	1.462 (4)	C5—H5	0.9300
C2—C3	1.352 (3)	C7—H7	0.9300
C2—C10	1.494 (3)	C8—H8	0.9300
C3—C4	1.418 (3)	C11—H11A	0.9700
C4—C5	1.404 (3)	C11—H11B	0.9700
C4—C9	1.393 (3)		
			
Br1···O3	2.9861 (19)	C5···O3^vi^	3.404 (3)
Br1···C11^i^	3.747 (3)	C6···C7^i^	3.569 (4)
Br1···H11A^i^	2.8900	C7···C6^v^	3.569 (4)
F1···O1^ii^	3.053 (3)	C8···C5^v^	3.574 (4)
F1···C8^ii^	3.226 (4)	C8···F1^iv^	3.226 (4)
F1···C9^ii^	3.259 (3)	C9···C4^v^	3.541 (3)
F1···H8^iii^	2.8400	C9···F1^iv^	3.259 (3)
O1···F1^iv^	3.053 (3)	C10···C1^i^	3.431 (4)
O2···C11	2.772 (3)	C10···O2^i^	3.230 (3)
O2···C2^v^	3.411 (3)	C11···Br1^v^	3.747 (3)
O2···C10^v^	3.230 (3)	C11···O2	2.772 (3)
O3···C2^i^	3.403 (3)	C1···H7^vii^	3.0600
O3···C3^vi^	3.296 (3)	C1···H11A	2.8800
O3···C5^vi^	3.404 (3)	C1···H11B	2.9200
O3···Br1	2.9861 (19)	C11···H11A^i^	3.1000
O1···H7^vii^	2.7600	H3···O3	2.4300
O2···H7^vii^	2.5200	H3···H5	2.5500
O2···H11B	2.5500	H3···O3^vi^	2.4500
O2···H11A	2.4400	H5···H3	2.5500
O2···H11B^v^	2.8500	H5···O3^vi^	2.6100
O3···H3	2.4300	H7···O1^iii^	2.7600
O3···H3^vi^	2.4500	H7···O2^iii^	2.5200
O3···H5^vi^	2.6100	H7···C1^iii^	3.0600
C1···C2^v^	3.420 (4)	H8···F1^vii^	2.8400
C1···C10^v^	3.431 (4)	H11A···Br1^v^	2.8900
C2···O2^i^	3.411 (3)	H11A···O2	2.4400
C2···O3^v^	3.403 (3)	H11A···C1	2.8800
C2···C1^i^	3.420 (4)	H11A···C11^v^	3.1000
C3···O3^vi^	3.296 (3)	H11B···O2	2.5500
C4···C9^i^	3.541 (3)	H11B···O2^i^	2.8500
C5···C8^i^	3.574 (4)	H11B···C1	2.9200
			
C1—O1—C9	123.43 (19)	C4—C9—C8	122.1 (2)
O1—C1—O2	116.5 (2)	O3—C10—C2	119.5 (2)
O1—C1—C2	116.7 (2)	O3—C10—C11	121.4 (2)
O2—C1—C2	126.9 (2)	C2—C10—C11	119.1 (2)
C1—C2—C3	119.4 (2)	Br1—C11—C10	113.15 (18)
C1—C2—C10	121.8 (2)	C2—C3—H3	119.00
C3—C2—C10	118.8 (2)	C4—C3—H3	119.00
C2—C3—C4	122.5 (2)	C4—C5—H5	121.00
C3—C4—C5	123.9 (2)	C6—C5—H5	121.00
C3—C4—C9	117.6 (2)	C6—C7—H7	120.00
C5—C4—C9	118.4 (2)	C8—C7—H7	120.00
C4—C5—C6	118.2 (2)	C7—C8—H8	120.00
F1—C6—C5	118.8 (2)	C9—C8—H8	121.00
F1—C6—C7	118.0 (3)	Br1—C11—H11A	109.00
C5—C6—C7	123.2 (2)	Br1—C11—H11B	109.00
C6—C7—C8	119.1 (3)	C10—C11—H11A	109.00
C7—C8—C9	119.0 (3)	C10—C11—H11B	109.00
O1—C9—C4	120.2 (2)	H11A—C11—H11B	108.00
O1—C9—C8	117.7 (2)		
			
C9—O1—C1—O2	−176.2 (2)	C3—C4—C5—C6	−177.3 (3)
C9—O1—C1—C2	2.9 (3)	C9—C4—C5—C6	0.7 (4)
C1—O1—C9—C4	2.0 (3)	C3—C4—C9—O1	−4.2 (3)
C1—O1—C9—C8	−178.5 (2)	C3—C4—C9—C8	176.3 (2)
O1—C1—C2—C3	−5.6 (4)	C5—C4—C9—O1	177.7 (2)
O1—C1—C2—C10	174.2 (2)	C5—C4—C9—C8	−1.8 (4)
O2—C1—C2—C3	173.4 (3)	C4—C5—C6—F1	−179.7 (2)
O2—C1—C2—C10	−6.8 (4)	C4—C5—C6—C7	0.6 (4)
C1—C2—C3—C4	3.6 (4)	F1—C6—C7—C8	179.5 (3)
C10—C2—C3—C4	−176.3 (2)	C5—C6—C7—C8	−0.8 (4)
C1—C2—C10—O3	−178.2 (2)	C6—C7—C8—C9	−0.4 (4)
C1—C2—C10—C11	0.2 (4)	C7—C8—C9—O1	−177.8 (2)
C3—C2—C10—O3	1.7 (4)	C7—C8—C9—C4	1.7 (4)
C3—C2—C10—C11	−179.9 (2)	O3—C10—C11—Br1	−3.2 (3)
C2—C3—C4—C5	179.4 (3)	C2—C10—C11—Br1	178.48 (18)
C2—C3—C4—C9	1.4 (4)		

Symmetry codes: (i) *x*+1, *y*, *z*; (ii) −*x*+1/2, *y*+1/2, −*z*+1/2; (iii) −*x*−1/2, *y*+1/2, −*z*+1/2; (iv) −*x*+1/2, *y*−1/2, −*z*+1/2; (v) *x*−1, *y*, *z*; (vi) −*x*+1, −*y*+1, −*z*; (vii) −*x*−1/2, *y*−1/2, −*z*+1/2.

### Hydrogen-bond geometry (Å, °)

**Table:**

*D*—H···*A*	*D*—H	H···*A*	*D*···*A*	*D*—H···*A*
C3—H3···O3^vi^	0.93	2.45	3.296 (3)	152
C7—H7···O2^iii^	0.93	2.52	3.425 (3)	164
C11—H11A···Br1^v^	0.97	2.89	3.747 (3)	148

Symmetry codes: (vi) −*x*+1, −*y*+1, −*z*; (iii) −*x*−1/2, *y*+1/2, −*z*+1/2; (v) *x*−1, *y*, *z*.
